# Deep Sclerectomy with Goniosynechiolysis Ab Interno for Chronic Glaucoma Associated with Peripheral Anterior Synechiae

**DOI:** 10.1155/2015/625719

**Published:** 2015-06-23

**Authors:** Alireza Mirshahi, Peter Raak, Katharina Ponto, Bernhard Stoffelns, Katrin Lorenz, Gábor B. Scharioth

**Affiliations:** ^1^Dardenne Eye Hospital, 53177 Bonn, Germany; ^2^Department of Ophthalmology, University Medical Center, Johannes Gutenberg-University, 55131 Mainz, Germany; ^3^Aurelios Eye Center, 45657 Recklinghausen, Germany; ^4^Department of Ophthalmology, University of Szeged, Szeged 6720, Hungary

## Abstract

*Purpose*. To report one-year results of phacoemulsification combined with deep sclerectomy and goniosynechiolysis ab interno for chronic glaucoma associated with peripheral anterior synechiae (PAS). *Methods*. We retrospectively analyzed medical charts of 16 patients (20 eyes) treated by one-site combined phacoemulsification and deep sclerectomy with goniosynechiolysis ab interno. PAS were transected by a spatula introduced into the anterior chamber through a paracentesis. To account for the correlation of right and left eyes a linear mixed model with unstructured covariance structure was calculated. *Results*. The mean preoperative intraocular pressure (IOP) was 20.3 ± 5.2 mmHg with 2.4 ± 1.0 medications. One year postoperatively, the mean IOP was 15.3 ± 3.3 mmHg (*P* = 0.004, paired *t*-test) with 0.6 ± 1.0 medications. A postoperative IOP of ≤21 mmHg without medication was achieved in 17 of 19 eyes (89.5%) and in 12/19 eyes (63.2%) at 3 and 12 months after surgery, respectively. In the remaining eyes (10.5% at 3 months and 36.8% at 12 months), additional medication led to an IOP ≤21 mmHg or the target pressure. No case required further glaucoma surgery. In one eye, conversion of the surgery to trabeculectomy was necessary due to Descemet's window rupture. *Conclusions*. With goniosynechiolysis ab interno, effective and safe nonpenetrating glaucoma surgery is possible in presence of PAS.

## 1. Background

Nonpenetrating glaucoma surgeries (NPGS), namely, deep sclerectomy and viscocanalostomy with or without implantation of surgical adjuvants, have been developed as an alternative to conventional filtering procedures [1–6]. These procedures aim to reduce the intraocular pressure (IOP) by enhancing the natural aqueous outflow channels; they reduce the outflow resistance at the inner wall of the Schlemm's canal and the juxtacanalicular trabecular meshwork [4, 7, 8]. The anterior chamber is not opened in the surgical field, thus reducing the likelihood of complications related to conventional penetrating glaucoma surgery (e.g., hypotony and choroidal detachment, among others) [3, 4, 9]. Although there is some inconsistency in comparing the efficacy of NPGS to trabeculectomy, there seems to be sufficient evidence that NPGS provides IOP reduction into the high teens [1, 10–12]. However, due to the nonpenetrating nature of these procedures, the presence of peripheral anterior synechiae (PAS) at the surgical area remains a challenge because the outflow through the Descemet's membrane is limited in presence of PAS. PAS can be treated during NPGS by a technique previously reported by our group [13]. By transecting the PAS during the surgery, spontaneous improvement of aqueous outflow through the trabeculo-Descemet's membrane has been observed. However, to date, no studies have assessed the clinical results of nonpenetrating glaucoma surgery and goniosynechiolysis ab interno in PAS-associated glaucoma.

This study was initiated and conducted to report the safety and efficacy of deep sclerectomy with goniosynechiolysis ab interno combined with phacoemulsification in the treatment of eyes with cataract and chronic glaucoma associated with PAS.

## 2. Methods

The protocol was consistent with the principles of the Declaration of Helsinki. Because of the retrospective nature of the study and because none of the individual-related data were passed to third parties, no approval by the Ethical Review Committee was required.

We retrospectively reviewed medical records of patients who had been subject to NPGS at our department between January 2000 and December 2006. Twenty eyes of 16 patients (8 female and 8 male, mean age 70.0 y, range 59–82) were identified who had been scheduled for a combined surgery that included NPGS, namely, deep sclerectomy with introduction of an adjuvant implant or ocular viscoelastic device (SK-Gel, T-Flux or Healon GV), phacoemulsification and posterior chamber intraocular lens (IOL) implantation (one-site surgery). The decision of goniosynechiolysis ab interno had been made intraoperatively for treatment of PAS. Choice of implant used was solely based on availability in the operating theatre. In all cases, the indication for the surgery was chronic glaucoma with peripheral anterior synechia (goniosynechiae) at the superior iridocorneal angle covering the trabecular meshwork and cataract. The indication for surgery was IOP above 20 mmHg under maximally tolerated medical therapy, or above 18 mmHg in cases of severe glaucomatous optic disc cupping (CDR above 0.8) or progression of the disease defined by mean deviation increase of greater than 2 db in the visual field testing. The cataract was clinically relevant in all eyes and surgical removal was considered useful. Patients with less than 12 months of follow-up were excluded. The following data were collected and analyzed: age, gender, pre- and postoperative IOP, pre- and postoperative glaucoma medication, type of glaucoma implant, and complications during and after the surgery.

### 2.1. Surgical Technique

**Figure 1:**
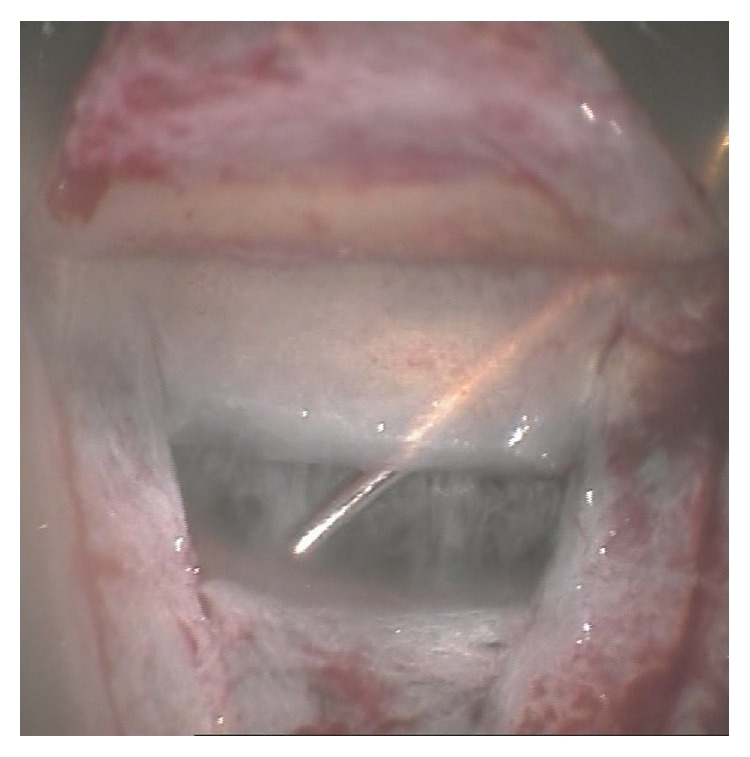
Goniosynechiolysis ab interno using a spatula introduced through paracentesis. Full visual control is attained through the peripheral trabeculo-Descemet's window.

The conjunctiva was opened carefully at the superior limbus using scissors. After minimal cauterization, a superficial scleral flap was created that reached far into the clear cornea. The superficial flap was sized 5 × 5 mm having a parabolic shape. The deep scleral flap had a triangular shape of approximately 3 × 3 × 3 mm. An incision of 2.8 mm width was made into the anterior chamber, and a tunnel was formed under the superficial flap. Phacoemulsification and IOL implantation were then performed. A deep scleral flap was prepared that was associated with an “unroofing” of Schlemm's canal and presentation of a “Descemet's window,” through which one could observe the anterior chamber and peripheral iris. Afterwards, the juxtacanalicular trabecular meshwork was peeled. The presence of peripheral anterior synechiae (goniosynechiae) in the surgical area usually prevents sufficient aqueous humor outflow, potentially leading to surgical failure. Under full visual control through the previously prepared “Descemet's window” (Figure 1), the anterior chamber was filled with air via paracentesis, followed by introduction of a fine iris spatula into the anterior chamber towards the surgical area. The air injection allows for stabilization of the anterior chamber. The inserted spatula is utilized to release the peripheral goniosynechia via a gentle swinging movement under direct visualisation through the trabeculo-Descemet's membrane. Air movement towards the iridocorneal angle, now open, confirmed successful goniosynechiolysis ab interno. The surgery was completed with the introduction of an implant (SK-Gel, T-Flux, or Healon GV) and watertight suturing of the superficial flap and conjunctiva. Further surgery was deemed necessary in the event that target IOP could be achieved with maximally tolerated medical therapy. Complete success was defined as IOP ≤21 mmHg without medication and qualified success with an IOP ≤21 mmHg with medication.

### 2.2. Data Collection and Analysis

All data was collected and stored by the same person (PR), using Microsoft Excel. The statistical analysis was performed with the program Bias for Windows (version 8.6.0, Frankfurt, Germany). In addition to descriptive statistical analysis, the paired *t*-test was applied for comparison of preoperative IOP versus IOP at 12 months after the surgery. The significance level was set at 0.05. To account for the correlation of the right and left eyes we performed a linear mixed model with unstructured covariance structure using SAS version 9.3.

## 3. Results

All surgeries were performed by the same experienced glaucoma surgeon (GS). With the described surgical technique, a selective treatment of peripheral anterior synechia was possible during NPGS by direct visualisation through a trabeculo-Descemet's window in 19 of 20 eyes (95%). In one case, the surgery was converted to phacotrabeculectomy with iridectomy due to intraoperative rupture of Descemet's window. This case was excluded from the final data analysis. All other operations were uneventful. To avoid secondary collapse of the superficial flap, SK-Gel (Corneal, Paris, France) was implanted in 10 eyes (50%), T-Flux (IOLTech, La Rochelle, France) in 6 eyes (30%), and Healon GV (AMO, Ettlingen, Germany) in 3 eyes (15%). Prior to surgery none of the eyes had a peripheral iridotomy. Preoperative gonioscopy had revealed interrupted areas of peripheral anterior synechiae (goniosynechiae) in all cases, and the angle was open (Shaffer II, III, or IV) in more than 180°. None of the eyes developed a pupillary block postoperatively. During the follow-up period no YAG laser goniopunctures were performed. No further glaucoma surgery was necessary in any of the cases during the follow-up period.

**Figure 2:**
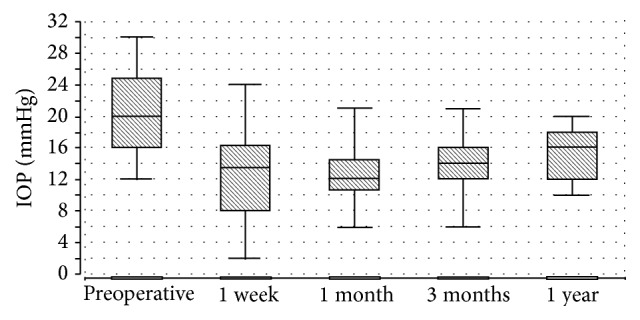
Course of the intraocular pressure after the surgery. Box plots indicate the minimum and maximum, as well as the 25% and 75% quartiles.

**Figure 3:**
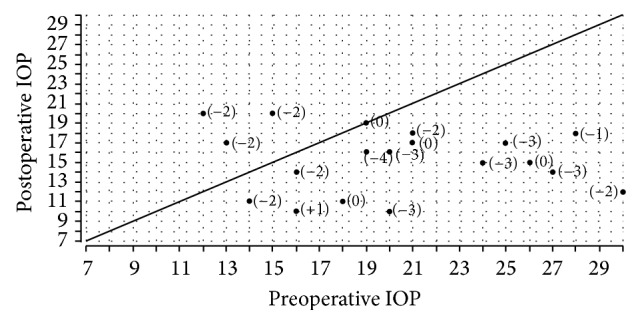
Scatterplot preoperative versus postoperative IOP. Change in number of medication in brackets.

The mean preoperative IOP was 20.3 ± 5.2 mmHg (median 20, range 12–30) on 2.4 ± 1.0 medications (median 2, range 0–5). The course of IOP was as follows (Figures 2 and 3): at day 1, mean IOP was 11.7 ± 6.1 mmHg (median 11, range 2–21); at week 1, mean IOP was 13.2 ± 6.7 mmHg (median 13.5, range 2–24); at month 1, mean IOP was 12.7 ± 3.5 mmHg (median 12, range 6–21); and at month 3, mean IOP was 14.2 ± 4.2 mmHg (median 14, range 6–21). One year postoperatively, the mean IOP was 15.3 ± 3.3 mmHg (median 16, range 10–20) with 0.6 ± 1.0 medication, which was significantly different from preoperative IOP (*P* = 0.004). Within the linear mixed model only time had an influence with the mean IOP being lowered by 5.96 mmHg, 95% confidence interval [2.83; 9.09], *P* = 0.001. This linear mixed model also showed that the results were not influenced by the inclusion of both eyes in 4 patients.

A postoperative IOP of ≤21 mmHg (complete success) was achieved in 17/19 eyes (89.5%) at 3 months and in 12/19 eyes (63.2%) at 12 months postoperatively. In the remaining eyes (10.5% at 3 months and 36.8% at 12 months), additional medication, at mean quantities of 0.3 and 0.6, respectively, led to an IOP ≤21 mmHg (qualified success). Using a more stringent criterion of IOP <18 mmHg, success was achieved in 15/19 eyes (78.9%) at three months and in 14/19 eyes (73.7%) at one year after surgery. One year after the surgery, the mean IOP reduction was 5.0 ± 6.4 mmHg (median 4, range −18 to +8). The mean number of medications decreased from 2.4 (median 2) before the surgery to 0.63 (median 0) one year postoperatively.

## 4. Discussion

Peripheral anterior synechia (goniosynechia), if present directly at the area of flap created in NPGS, may compromise the surgical outcome. Peripheral anterior synechia can lead to a potential failure of NPGS if they occur at the sites where NPGS would typically reduce the outflow resistance, namely, at the trabecular meshwork adjacent to the sclerocorneal flap. In a prospective study, Moreno-Montañés et al. found that goniosynechiae are associated with worse postoperative IOP control after deep sclerectomy [14]. Releasing these synechiae using the technique previously described by our group [13] not only may be primarily beneficial by clearing the outflow tract but also may be advantageous if Nd:YAG laser goniopuncture is needed postoperatively (in the case of insufficient IOP regulation after primary surgery). Absence of PAS enables the laser surgeon to aim directly for perforation of Descemet's membrane with a Nd:YAG laser without any obstruction, thus leading to a lower risk of inadvertent laser damage to the peripheral iris, which in itself would induce further goniosynechiae after the Nd:YAG goniopuncture. In our experience, releasing the PAS by this maneuver led to an immediate improvement of aqueous humor outflow through the trabecular meshwork. Although we cannot quantify this improvement, we believe that treatment of PAS, when present at the surgical field, enhances the trans-descemetic outflow in NPGS. In our series the surgery was completed with the introduction of an implant (SK-Gel, T-Flux, or Healon GV). The SK-Gel and the Healon GV are implants that dissolve over time in about 4 to 6 months after surgery, as they are made of hyaluronic acids. On the other hand the T-Flux is a nonabsorbable implant made of Poly Megma (hydrophilic acrylic). The overall results and success rates might differ upon absorption or nonabsorption of the implant. Our study sample is too small to answer to this question and this is certainly a limitation. Furthermore, the follow-up time is limited; thus we are not able to provide long-term results of our study sample. The presence of goniosynechia might be related to a previous intraocular inflammation. In our study sample the patients did not report previous inflammatory disease and no active inflammation was detectable at the time of presentation. A preoperative gonioscopy had revealed presence of very few goniosynechia; however the cases have been classified as open angle glaucoma, as inflammation was neither reported nor present, and almost all parts of the angle were clearly open. The functional relevance of the goniosynechia at the surgical field was detected during the surgery. As a matter of fact this is the case in a very small group of patients.

Schreyger et al. report a mean reduction of IOP of around 5.5 mmHg with reduction of medications from 2.0 preoperatively to 0.3 after deep sclerectomy and phacoemulsification [15]. The type of implant used (SK-Gel versus T-Flux) does not seem to have an impact on the success of the surgery [15]. In a meta-analysis, Cheng et al. conclude that the efficacy of nonpenetrating glaucoma surgery as measured by IOP reduction is equivalent in cases with and without introduction of an implant during the surgery [16]. Our results are similar to those reported by Schreyger et al.; we noted a mean IOP reduction of 5.0 ± 6.4 mmHg and a reduction in the number of medications from 2.4 before surgery to 0.6 after surgery. The IOP reductions in both studies fall within the same range. It is also noteworthy that the operations in both studies were performed by the same surgeon (GS). We, thus, conclude that the IOP reduction seen in eyes with chronic glaucoma and PAS treated with NPGS with goniosynechiolysis ab interno in combination with phacoemulsification is similar to the IOP reduction seen in eyes with POAG without PAS treated with NPGS and phacoemulsification.

The exact position of the peripheral anterior synechiae can be determined prior to NPGS by gonioscopy, and the surgeon may choose to create a flap at another position (e.g., lateral) where such adhesion may not be present. In our experience, however, this situation is rare as goniosynechiae are often present at several positions around the iridocorneal angle. As a result, changing the access location is not always an option. Moreover, most surgeons perform this very delicate surgery from a preferred access area (usually superior) and changing this preferred access site may lead to a worse outcome.

Some surgeons make microincisions in Descemet's membrane (Descemet's fenestration) if the outflow test reveals insufficient aqueous flow during the surgery. This technique may also be an option in the presence of PAS. However, in our experience, synechiolysis ab interno as performed in our series of patients unequivocally enhances outflow, which can be directly visualized during the surgery [13]. Descemet's fenestration may be considered if goniosynechiolysis does not lead to the desired outflow rate.

In the case that the spatula inadvertently damages and perforates the trabeculo-Descemet's membrane during goniosynechiolysis, the iris should be observed carefully. If there is an iris prolapse, a peripheral iridectomy should be performed; if there are only microperforations without iris prolapse, injection of air into the anterior chamber can be considered because air maintains the anterior chamber and prevents iris incarceration into the defect of descemetic membrane. In our experience, microperforations occurred occasionally after the release of the PAS by the described technique. Surgery can and should be converted to trabeculectomy in case of Descemet's membrane rupture and iris prolapse.

The efficacy and success rates of NPGS, in terms of IOP reduction, do not appear to be compromised when combined with phacoemulsification and intraocular lens implantation [4]. D'Eliseo et al. reports a higher success rate (IOP ≤20 mmHg without medication) when combining deep sclerectomy with phacoemulsification versus deep sclerectomy alone (90% versus 62%) [17], whereas Marek et al. report similar success rates when comparing phacodeep sclerectomy and deep sclerectomy alone [18]. Thus, we conjecture that the combination of phacoemulsification and deep sclerectomy surgery most likely does not compromise the success rate with regard to IOP reduction. In fact, it is possible that cataract surgery exerts a positive influence on the postoperative IOP. However, it seems unlikely that the cataract surgery alone would have led to the IOP reduction seen with the combined surgery. We do not have data on a control group; however we believe that reduction of the preoperative IOP from 20.3 ± 5.2 mmHg with 2.4 ± 1.0 medications to 15.3 ± 3.3 mmHg with 0.6 ± 1.0 medications after the surgery appears to be impossibly attributed to the phacoemulsification alone.

Phacoemulsification and intraocular lens implantation were performed at the same surgical area as the deep sclerectomy (superior, one-site surgery). In a literature review (January 2012), we were unable to find any comparative studies on one-site versus two-site combined NPGS and phacoemulsification. Therefore, it remains unclear whether the cataract surgery incision site had any influence on safety or efficacy of the procedure in our study. Similarly, there were no Medline-listed publications on the efficacy of trabeculectomy in presence of peripheral anterior synechia. Although we do not expect the presence of PAS to compromise the efficacy results of trabeculectomy, no published study data are available supporting this expectation.

Our positive experience with goniosynechiolysis ab interno, as presented in this paper, has encouraged us to perform nonpenetrating glaucoma surgery even in the presence of peripheral anterior synechiae. After development and publication of the goniosynechiolysis method our study delivers first data on the efficacy and safety of this procedure. A comparative study of nonpenetrating glaucoma surgery with goniosynechiolysis ab interno versus conventional trabeculectomy in presence of peripheral anterior synechia would shed light on the question of comparative efficacy and safety of these methods.

## 5. Conclusions

With goniosynechiolysis ab interno, the surgeon is able to perform nonpenetrating glaucoma surgery even in presence of peripheral anterior synechia. Deep sclerectomy with goniosynechiolysis ab interno in combination with phacoemulsification was safe and effective in treatment of eyes with cataract and chronic glaucoma associated with peripheral anterior synechia.
